# The impact of industry-university-research projects on biopharmaceutical companies’ innovation performance: moderating roles of government subsidies for innovation

**DOI:** 10.3389/fpubh.2023.1271364

**Published:** 2023-11-15

**Authors:** Yuntian Xia, Yiwen Jia

**Affiliations:** ^1^School of Economics and Management, Hefei Normal University, Hefei, China; ^2^School of Management, University of Science and Technology of China, Hefei, China; ^3^Department of Gastroenterology, Hefei First People’s Hospital, Hefei, China; ^4^Department of Gastroenterology, The Third Affiliated Hospital of Anhui Medical University, Hefei, China

**Keywords:** industry-university-research cooperation, innovation, quality, inputs, innovation subsidies

## Abstract

Innovation holds paramount importance for both nations and businesses. This article presents a panel regression model designed to assess the fixed effects of industry-university-research (IUR) cooperation projects on innovation performance. Furthermore, it examines the moderating impact of government innovation subsidies by utilizing data spanning from 2007 to 2021, encompassing 326 listed Chinese biopharmaceutical firms. Our findings reveal that industry-university-research-cooperation projects have the potential to significantly enhance innovation performance across three key metrics: input, output, and quality for firms. The presence of government innovation subsidies as a moderator is found to have a positive influence on IUR-cooperation projects and their innovative inputs. However, it can yield adverse effects on IUR-cooperation projects with respect to innovation outputs and quality. The insights presented in this paper introduce innovative recommendations for elevating corporate innovation quality and refining the policies governing IUR cooperation.

## 1. Introduction

The biopharmaceutical industry is one of the high-tech sectors that showcase the strength of the national economy and is closely tied to the population’s health. The innovation performance of the biopharmaceutical industry not only yields economic benefits but also contributes to long-term health advantages. The widely adopted approach known as industrial-university-research (IUR) cooperation involves establishing robust partnerships with universities, research institutes, and companies. This strategy is particularly notable within the biopharmaceutical industry, which operates in a highly competitive and increasingly complex environment (1). In the context of an economy’s innovation system, universities and research institutes play a pivotal role as pioneers in knowledge advancement, serving as the primary source of technological innovation and talent cultivation (2). Firms bridge the gap between technology development and market demand, converting scientific knowledge into practical productivity (3).

China’s growing emphasis on science and technology innovation has led to remarkable progress in the development of breakthroughs, with the number of patent applications ranking at the top globally. Industry-university-research, as a novel approach to fostering innovation (4, 5), has also been progressively integrated into policy. In our search for keywords related to IUR, we scoured the websites of various ministries and commissions, including the Chinese government website, the Ministry of Science and Technology, the Ministry of Education, and the Ministry of Industry and Information Technology, as well as Peking University’s Legal Information Network. To date, there have been approximately 2,000 or more policy documents on IUR-cooperation and cooperative innovation at the national level, including the Central Committee of the Communist Party of China, the State Council, the National People’s Congress, and the ministries and commissions under the State Council, until 2022. These policy documents come in 25 different forms, encompassing laws, rules, opinions, plans, notices, programs, and blueprints, among others. Based on our data, the number of cooperation projects established between listed companies in China’s biopharmaceutical industry and universities, or research institutes is significantly higher than the average for all listed companies. Simultaneously, the Chinese government is keen on promoting the development of self-developed pharmaceuticals, leading to extensive policy support. Hence, it is reasonable to select the biopharmaceutical industry as the focus of this study.

According to the research objectives, the literature on the relationships between IUR-cooperation and innovation can be categorized into three main groups. The first category of literature takes a macro approach to evaluate the intensity, sustainability (6), participation of each subject (7), influencing factors (8), and regional innovation performance of IUR-cooperation, the first category of literature adopts a macro approach (9, 10). The second category of literature focuses on universities, aiming to assess their role, performance, and the influencing factors in industry-university cooperation (11, 12), suggests that researchers engaged in industry-university cooperation tend to produce more and higher-quality articles. Additionally, TurkBicakci et al. (13) examine the impact of university participation in industry-university cooperation, considering factors such as institutional status, the nature of the institution, and research intensity (14). The third category of literature concentrates on the performance of firms, exploring topics like the objectives and benefits of enterprise participation, the challenges they face, and the criteria they employ when selecting cooperation partners (15, 16). Numerous studies employ publications and patents as significant indicators of IUR-cooperation. However, it is important to note that innovation is a high-risk activity, where the intention to cooperation comes first, and the results are produced second. Within this context, several important and relevant questions deserve our attention. For example, what is the impact of IUR-cooperation projects on firms’ innovation performance? As the government progressively promotes the cooperative innovation model, does it moderate IUR cooperation and the innovation performance of firms?

Bases on the knowledge-based theory of the firm (17, 18), firms integrate the specialist knowledge of their member. This entails a complex web of coordination both within and beyond the boundaries of these firms. This efficient integration is achieved through cross-learning among organizational members. Furthermore, universities and research institutions possess their unique reservoirs of specialized knowledge and can serve as crucial partners and coordinators in cooperation with these firms. Government can significantly enhance a company’s knowledge and learning capabilities, fostering the creation, and sharing of organizational knowledge. Government intervention aids firms in better aligning their strategies. We have undertaken the manual collection of IUR-cooperation projects within the biopharmaceutical industry, and we employ three distinct criteria to characterize corporate innovation performance, namely inputs, outputs, and quality. This article presents a panel regression model designed to assess the impact of IUR-cooperation on innovation performance, while also evaluating the moderating effect of government innovation subsidies. The data used in our analysis is drawn from 326 publicly listed Chinese biopharmaceutical firms, spanning the years 2007 to 2021. Furthermore, our study investigates the influence of government innovation subsidies on the relationship between IUR-cooperation and innovation performance. To ensure the robustness of our findings, this research employs several rigorous checks, including variations in dependent variables and instrumental variable analysis.

Overall, this work contributes significantly to current research in four keyways. Firstly, it provides a formal and rigorous empirical analysis of IUR-cooperation, addressing the existing dearth of such studies. Much of the existing literature on IUR-cooperation relies on theoretical analysis or questionnaire-based results (19, 20), some of which are subjective in nature. Moreover, many articles discussing IUR primarily focus on the number of patents (21), often overlooking the fact that cooperative projects precede patent filings for publicly traded companies. Research and development processes are intricate and demanding; therefore, enterprises, whether in collaboration with universities or research institutes, embark on these projects with vital research objectives. Additionally, the innovation of Chinese pharmaceutical companies has been historically characterized by a focus on imitation over originality and a tendency to prioritize quantity over quality. When evaluating the innovation performance of the pharmaceutical industry, the quality of innovation, as reflected in the ratio of exploratory patents, emerges as a crucial metric. Lastly, prior studies examining the factors influencing IUR-cooperation have largely considered the perspectives of companies, research institutions, and the broader open environment (22). However, increased government emphasis on the significance of IUR-cooperation, a substantial portion of government involvement has become apparent. In previous literature, the government’s perspective was notably absent.

The rest of this essay is organized as follows. Section 2 provides a literature review and outlines the research hypotheses. In Section 3, we describe the models, variables, and data. Section 4 covers the empirical findings, including several robustness tests, and presents descriptive statistics of the variables. Finally, Section 5 concludes the paper.

## 2. Theoretical background and hypotheses development

Based on the knowledge-based view, the stages of corporate knowledge generation do not solely rely on internal development but also necessitate cooperation with institutions such as universities and research organizations to jointly develop commercial knowledge, share risks, and reap mutual benefits (23). IUR-cooperation represents a diverse integration of knowledge between firms and universities or research organizations (24). Within this cooperation, universities and research institutes focus on fundamental research, while businesses concentrate on product development (25). Universities and institutions bring forth extensive complementary and diverse expertise (26),much of which is tacit, requiring interpersonal communication for knowledge integration (27). China has several IUR projects, including cooperation between universities and research institutes through R&D institutions affiliated with enterprises and strategic alliances between universities and businesses (28). In these projects, professionals from firms, universities, and research institutions collaborate on various levels (29). Universities, research institutes, and firms act as the production and input sides of knowledge, respectively, applying this knowledge to various aspects of innovative activities within the industry community through the knowledge flow process (30). The dissemination of knowledge from universities and research institutions to businesses is facilitated by formal or informal interactions between firms and these institutions (31).

### 2.1. The impact of industry-university-research-cooperation projects on firms’ innovation input

The innovation process involves strategic efforts aimed at acquiring a diverse range of knowledge necessary for fostering innovation (32). Companies often opt to engage in cooperative partnerships with universities and research institutes, harnessing their technical and academic expertise to advance cutting-edge technologies and ideas that align closely with the company’s specific objectives, as opposed to pursuing independent development (33). This cooperative approach tends to yield significant benefits, prompting stakeholders to consider augmenting their investments in R&D (34). As a result, these companies can engage more effectively with research institutes to efficiently access novel products and technologies. Such cooperative initiatives also serve to underscore a company’s robust R&D capabilities, which, in turn, can enhance their attractiveness to potential investors. This heightened investor interest can ameliorate the financing challenges faced by listed companies to a certain extent. Consequently, listed companies often demonstrate a proclivity for entering into cooperative agreements with universities and research institutes and disclosing these partnerships. This strategic cooperation not only provides access to a wider pool of capital for innovation investments but also bolsters their innovation efforts through enhanced resources and expertise. Thus, we propose the following hypothesis:

*H1a*: Industry-university-research-cooperation projects lead to innovation inputs.

### 2.2. The impact of industry-university-research-cooperation projects on firms’ innovation output

Acquiring knowledge and skills through cooperation have been effective and efficient means of successful innovation (35). In pursuit of optimizing their innovation investments and expediting the commercialization process, companies tend to accord precedence to research initiatives that are characterized by low levels of risk and complexity (36). In this regard, efforts within the sphere of IUR-cooperation offer a distinct advantage, being both cost-effective and less fraught with risk when compared to internal R&D. This cost-effectiveness stems from the capacity of companies to leverage the existing scientific expertise and equipment available within universities and research institutions, thus circumventing the substantial expenditures associated with personnel recruitment and equipment procurement (37). Moreover, knowledge sharing is a mechanism to convert tacit into explicit knowledge (38). Industry-university-research cooperation projects facilitate the acquisition and integration of inter-organizational resources, including research talent, equipment, and facilities (39). Universities and research institutions can offer valuable technical guidance to businesses (40). This approach not only aids companies in overcoming technical challenges and accelerating R&D but also assists in resolving a range of issues, thus fostering innovation. As a result, through IUR-cooperation, organizations can effectively reduce restrictions on R&D expenditure and mitigate innovation risks while simultaneously enhancing their innovation outputs. Consequently, we propose the following hypothesis:

*H1b*: Industry-university-research-cooperation projects lead to innovation outputs.

### 2.3. The impact of industry-university-research-cooperation projects on firms’ innovation quality

Open innovation represents an agile innovation process that involves the assimilation of both internal and external knowledge and technologies through cooperative relationships (41). IUR-cooperation projects confer a distinct advantage upon companies by harnessing their strengths in basic research, thereby fostering the development of groundbreaking and original innovations. Owing to differences in market orientations and resource constraints (42), businesses frequently channel their efforts toward realizing incremental advancements in low-risk, cost-effective applications (43). The overarching objective is to expedite the transformation of these innovations into tangible economic value (44). In contrast, universities and research institutes primarily focus on fundamental research with an emphasis on breakthrough-oriented innovation (45), leading to fundamentally rooted innovation outcomes (46). Universities and research organizations can provide businesses with well-qualified technical guidance (47). This approach supports companies in innovating, aiding them not only in addressing technical challenges but also in resolving a diverse range of issues. Industry-university-research cooperation projects can, therefore, divert businesses from their path of solely incremental innovation, empowering them to attain exploratory innovation performance by drawing upon the fundamental research of universities and research institutions. Based on this discussion, we propose the following hypothesis:

*H1c*: Industry-university-research-cooperation projects lead to innovation quality.

### 2.4. The moderating role of government innovation subsidies

The government plays a crucial role by providing financial support, knowledge about cutting-edge technologies, and the latest industry policies. Incorporating this type of knowledge is of significant benefit to firms (48). Business and government ties lead to both economic and operational performance (49).

Government innovation subsidies, on one hand, by removing financial barriers to industry-university-research (IUR) cooperation projects, can enhance the innovative input performance of enterprise (50), but also confirm the direction of innovation. IUR-cooperation projects aim to mitigate the costs and risks of innovation to enhance innovation performance (51). Subsidies can serve as financial support for firms’ innovation initiatives and signal a commitment to acquiring additional societal resources (52). As a result, government innovation subsidies can significantly alleviate the financial constraints faced by companies engaged in IUR-cooperation (53), enabling them to offer high-quality innovative inputs and contribute to the successful implementation of projects involving industry, academia, and research.

On the other hand, the existing innovation support strategy is primarily based on the assessment of firms’ R&D and innovation investments, as well as patent applications (54). Government innovation subsidies encourage firms to pursue short-term goals in IUR-cooperation. However, high-quality innovation often requires longer periods of R&D investment. Still, government officials, under pressure for performance assessment, tend to select innovative projects with short timelines and quick outcomes for their innovation support policies (55). This can lead to firms’ preference for short-term goals in IUR-cooperation. They may be more inclined to select cooperation projects that can yield rapid inventive outcomes, which can be detrimental to their innovation outputs and quality (22). Moreover, after receiving innovation subsidies, companies may focus on short-term innovation objectives (56). Larger government subsidies may encourage opportunistic behavior among firms (57). Since it is challenging for the government to effectively assess the genuine quality of innovation achievements when selecting funding recipients, when government subsidies are increased, firms are more likely to pursue low-complexity and low-level innovation projects in IUR-cooperation. These firms may prioritize low-quality innovative outputs to access subsidized funding (58). This effect, known as “bad money drives out good,” results in the abandonment of genuinely beneficial IUR-cooperation initiatives, making it challenging for firms to gain government support. Consequently, this undermines the positive impact of IUR-cooperation projects on enhancing innovation output and quality.

The following competing hypotheses are put up for more empirical investigation in this work based on the analyses presented above:

*H2*: Government innovation subsidy positively moderates the relationship between IUR-cooperation projects on firms’ innovative input.

*H3*: Government innovation subsidy negatively moderates the relationship between IUR-cooperation projects on firms’ innovative output.

*H4*: Government innovation subsidy negatively moderates the relationship between IUR-cooperation projects on firms’ innovative quality.

**Figure 1:**
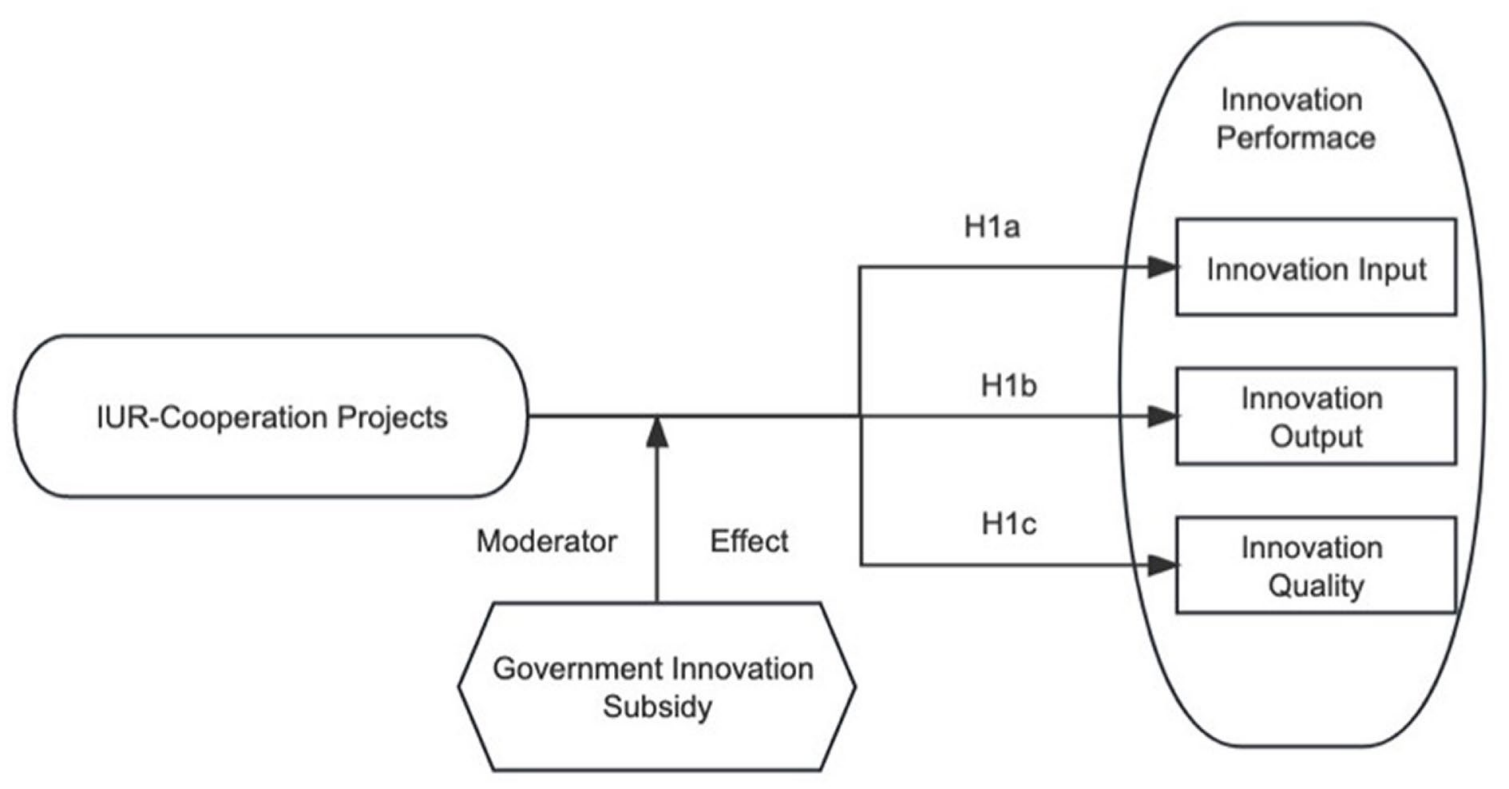
Conceptual framework.

Combining the above hypotheses, the conceptual framework of the study is shown in Figure 1.

## 3. Research methodology

### 3.1. Sample selection and data sources

To mitigate the impact of significant accounting standard revisions, this paper utilizes data from biopharmaceutical companies listed on A-shares. After excluding companies subjected to special treatment during the observation period, those operating in the financial and insurance sectors, companies issuing both B shares (foreign capital shares) and H shares (listed in Hong Kong), and companies with significant missing data, the final research sample comprised 326 companies. Financial data from these firms spanning the years 2007 to 2021 were sourced from the CSMAR database. The patent application data presented in this research were sourced from the website of the State Intellectual Property Office of China. By using the website’s patent examination function, we collected data on nearly one million patent applications submitted by all listed companies during the sample period. Furthermore, we manually compiled information regarding IUR-cooperation projects from publicly traded company disclosures. To ensure comprehensive control over all macro and micro factors influencing firms’ decisions on IUR-cooperation, this study encompasses not only enterprise-level variables, but also macro-level statistics derived from the WIND database specific to the regions where these enterprises are located. The variables have been scaled to the 1 and 99% percentiles to mitigate the influence of outliers.

### 3.2. Variables

#### 3.2.1. Dependent variables

This article examines three aspects of Innovation: inputs, outputs and quality (59, 60). Innovation **input** is assessed using established practices outlined in prior literature. Two key indicators are employed: the ratio of annual R&D expenditure to total assets (RD1) and the ratio of annual R&D expenditure to operating revenue (RD2). Innovation **output**, on the other hand, is evaluated by considering the number of patent applications submitted by the applicant company. These patents can be categorized based on the China Patent Classification, including invention patents, utility model patents, and design patents. Utility model patents pertain to new technical solutions involving shapes, structures, or their combinations that are practically applicable. Design patents relate to novel designs encompassing shapes, patterns, or their combinations, along with combinations of colors and shapes or patterns that exhibit esthetic appeal and suitability for industrial use. Invention patents are considered the most innovative, followed by utility model patents and design patents. To measure innovation output, this paper adopts two indicators following the approach of Bereskin et al. (61). (1) Patent1 represents the natural logarithm of the sum of the three patent categories plus one. (2) Patent2 accounts for the varying contribution weights of the three types of patents, subjectively assigning a 3:2:1 weight distribution. It is expressed as the natural logarithm of the weighted total number of the three types of patents plus one (62, 63). Patent1 is used for the principal model evaluation, while patent2 is applied in the robust test. This research identifies invention patents, characterized by high R&D complexity and innovation levels, as indicative of high-quality innovation outcomes, in line with the insights of Rong, Wu and Boeing (64) and other studies. A greater number of invention patents acquired by a firm signifies a higher quality of innovation. We employ the proportion of exploratory patents as an indicator of a company’s **quality**. The exploratory patent metric (65) defines a patent as exploratory if 60% or 80% of the patent classification numbers cited in the patent are unrelated to the firm’s existing patent portfolio. The Qua1 is computed as the ratio of 60% unrelated patent citations to the total number of innovations and inventions. Qua2 represents the ratio of 80% of patent citations unrelated to existing patents to the total number of patent citations. If a company’s Qua1 value is predictive, it suggests that its innovations are of higher quality. Qua1 is utilized in the primary regressions, while Qua2 is applied in the robust tests.

#### 3.2.2. Independent variables

In this study, the primary method for collecting data on Industry-University-Research (IUR) cooperation projects is manual collection. We used various search terms such as “university,” “research institute,” “industry institute,” “cooperation program,” “cooperative research and development,” and “joint research and development” to source documents from listed biopharmaceutical companies. These documents include the company’s annual reports, public records of board meetings, social responsibility reports, and other disclosures. We then compiled statistics based on these disclosures. For instance, if publicly traded company A discloses IUR activities with university X, we count this as ONE IUR cooperation for company A in the current year. Some publicly traded companies disclose both the amount and number of projects, while others simply state that they are cooperating with a specific university or institute. Our data collection and statistical compilation follow the aforementioned method. In this research, we utilize an approach developed by Hong and Su (66) and Park, Hong and Leydesdorff (67), to assess the natural logarithm of the number of cooperative IUR projects. The larger this variable, the higher the proportion of businesses participating in IUR-cooperation projects, and the more substantial this cooperation is in the context of their overall innovation activities.

#### 3.2.3. Moderators

The moderator is government innovation subsidies (GIS). In the notes to listed companies’ annual reports, we look for government grants. Keywords for the screening criteria include: “first set,” “science and technology support program,” “standardization strategy,” “research and development,” “development,” “innovation,” “science and technology,” “technology development,” “technology project grant,” “significant technology application,” “productivity promotion centre,” “incubator,” and “Golden Sun.” Following that, keywords are made as patents, copyrights, new products, and intellectual property rights. Novel cancer therapies, spores, antibiotics, and other forms of biological medical technology are studied as filters. Finally, we determine the total annual innovation subsidies granted to each listed company. We calculate as the natural logarithm of government innovation subsidies plus 1 (68).

#### 3.2.4. Control variables

To control as much as possible for each contributing element of firm innovation quality and to prevent endogeneity difficulties caused by neglecting essential factors, we control for both firm-level and regional-level variables of the province where the firm’s office is located. The firm-level control variables include Size (natural logarithm of assets), Lev (ratio of total debt to total assets), Roa (return of assets),ATO (Total Asset Turnover), Cash(Cash Flow Ratio), REC (Percentage of accounts receivable), Cur (Current Ratio), FIXED (ratio of fixed assets). The amount of control variables for the regional layer surface includes: col. (Number of high school projects in each province), Pgdp (gdp *per capita*), pop (Population size of each province) (69, 70).

### 3.3. Models


(1) $$\mathrm{Innovatio}n_{i,t}=\beta_{0}+\beta_{1}\mathrm{IU}R_{i,t}+\mathrm{\beta Contro}l_{i,t}+\alpha_{i}+\alpha_{t}+\varepsilon_{i,t}$$

where $\mathrm{Innovatio}n_{i,t}$ is the degree of company innovation, $\mathrm{IU}R_{i,t}$ is the level of IUR-cooperation projects, $\mathrm{Contro}l_{i,t}$ is a set of firm-level and regional-level control variables presented in this study, $\alpha_{i}$ is an individual fixed effect, $\alpha_{t}$ is a period fixed effect, and $\varepsilon_{i,t}$ is a random error term.


(2) $$\begin{array}{l} \mathrm{Innovatio}n_{i,t}=\beta_{0}+\beta_{1}\mathrm{IU}R_{i,t}+\beta_{2}GIS_{i,t}+\beta_{3}\mathrm{IU}R_{i,t}\\ \;\times GIS_{i,t}+\mathrm{\beta Contro}l_{i,t}+\alpha_{i}+\alpha_{t}+\varepsilon_{i,t} \end{array}$$

Equation (2) displays the empirical model used to investigate the moderating influence of government innovation subsidies. $GIS_{i,t}$ is indicators of government innovation subsidies (71).The interaction term between the amount of IUR-cooperation projects and the government innovation subsidy is denoted by $\mathrm{IU}R_{i,t}\times GIS_{i,t}$ .

## 4. Empirical results

### 4.1. Descriptive statistics and correlations

The descriptive statistics for all relevant variables are shown in Table 1. The average value of the natural logarithm IUR is 1.861, and the standard deviation is 1.409. These statistics indicate that IUR-cooperation projects are still at a modest level but exhibit significant heterogeneity. RD1, Patent1 and Qua1 have respective means (medians) of 0.017(0.012), 2.246 (2.303), and 0.267(0.427). This result implies that the average inputs and outputs of innovation have increased over the sample period. Other variable distributions are identical to those reported in prior studies.

**Table 1:** Descriptive statistics.

Variable	N	Mean	SD	Median	Min	Max
RD1	2,941	0.0170	0.0190	0.0120	0	0.0920
Patent1	2,941	2.246	1.736	2.303	0	6.518
Qua1	2,941	0.227	0.267	0.427	0	0.916
GIS	2,941	1.627	4.177	0	0	15.66
IUR	2,941	1.861	1.049	2.079	0	3.738
Size	2,941	22.05	1.289	21.87	19.70	26.06
Lev	2,941	0.426	0.208	0.419	0.0500	0.894
ROA	2,941	0.0430	0.0640	0.0410	−0.230	0.220
ATO	2,941	0.662	0.454	0.560	0.0670	2.645
Cashflow	2,941	0.0460	0.0720	0.0460	−0.177	0.246
REC	2,941	0.116	0.102	0.0920	0	0.460
INV	2,941	0.149	0.139	0.114	0	0.719
FIXED	2,941	0.217	0.164	0.183	0.00200	0.710
col	2,941	10.53	0.872	10.69	0	11.71
pgdp	2,941	10.73	0.568	10.72	8.959	12.12
pop	2,941	17.76	0.673	17.91	14.88	18.65

Table 2 illustrates the Pearson correlations among the variables, with the majority being significant but tiny. Industry-university-research-cooperation projects are strongly positively connected with RD1, Patent1 and Qua1, preliminarily indicating that IUR-cooperation projects can greatly boost innovation inputs and outputs. As a result of the fact that all VIFs are below the 10-point threshold (72, 73), there are no obvious correlations between variables. A Hausman test also shows that a fixed effect model should be employed in this investigation. The majority of indexes have been analyzed in accordance with existing research, and only significant variables have been integrated into our models as control variables (74).

**Table 2:** Pearson’s correlation matrix for all variables.

	RD1	Patent1	Qua1	IUR	GIS	Size	Lev	ROA	ATO	Cashflow	REC	INV	FIXED	col	pgdp	pop	VIF
RD1	1																
Patent1	0.391***	1															
Qua1	0.416***	0.927***	1														
IUR	0.114***	0.184***	0.190***	1													
GIS	0.080***	0.114***	0.184***	0.190***	1												1.05
Size	−0.043***	−0.171***	0.292***	0.191***	0.012**	1											1.51
Lev	−0.084***	−0.271***	0.018***	−0.040***	−0.035***	0.482***	1										2.01
ROA	0.048***	0.156***	0.075***	0.071***	0.021***	−0.031***	−0.393***	1									1.51
ATO	−0.00600	0.074***	0.068***	0.038***	0.011**	0.033***	0.132***	0.176***	1								1.14
Cashflow	−0.012**	0.080***	0.035***	0.019***	−0.012**	0.058***	−0.150***	0.363***	0.121***	1							1.34
REC	0.144***	0.350***	0.273***	0.293***	0.102***	−0.183***	−0.008	−0.011**	0.152***	−0.191***	1						1.34
INV	−0.030***	−0.140***	−0.082***	−0.096***	−0.027***	0.106***	0.320***	−0.086***	0.032***	−0.229***	−0.098***	1					1.40
FIXED	−0.079***	−0.187***	−0.067***	−0.088***	−0.019***	0.098***	0.108***	−0.111***	0.00800	0.239***	−0.283***	−0.307***	1				1.44
col	0.029***	0.285***	0.225***	0.237***	0.051***	0.053***	−0.116***	0.049***	0.00100	0.021***	0.196***	−0.037***	−0.204***	1			1.64
pgdp	−0.042***	0.084***	0.026***	0.018***	−0.032***	0.071***	−0.023***	−0.00800	−0.028***	−0.00800	0.047***	−0.028***	−0.118***	0.237***	1		1.34
pop	0.099***	0.120***	0.138***	0.148***	0.080***	−0.079***	−0.059***	0.047***	0.060***	0.037***	0.092***	−0.026***	0.009*	0.424***	−0.301***	1	1.59

Standard errors in parentheses. ***p < 0.01, **p < 0.05, *p < 0.1.

### 4.2. Main regression analysis

The empirical results of the effect of IUR-cooperation projects on innovation performance can be found in Table 3. Column (1) shows the regression R&D inputs for model (1). The coefficient of the IUR-cooperation projects is 0.0002 which is highly significant at the 0.01 level (*t* = 0.0000). This finding demonstrates that IUR-cooperation projects have positive importance with R&D inputs. Column (2) displays the findings following the addition of several control factors, suggesting that IUR-cooperation projects remain significant despite considering the endogenous difficulties produced by the missing variables. The results of estimating innovation outputs without and with control variables are presented in columns (3) and (4), respectively. Industry-university-research-cooperation’s principal coefficients are 0.0419 and 0.0149, which is statistically positive at the 0.01 and 0.1 level. The findings of evaluating innovation quality without and with control variables are presented in columns (5) and (6), respectively. Significantly positive at the 0.01 level are the coefficients 0.0029 and 0.0017 for the IUR-cooperation. This result suggests that IUR-cooperation projects can increase innovation inputs, outputs, and quality for businesses. These data thus support H1.

**Table 3:** Empirical results of the impact of IUR-cooperation projects on innovation performance.

	(1)	(2)	(3)	(4)	(5)	(6)
Variables	RD1	RD1	Patent1	Patent1	Qua1	Qua1
IUR	0.0002**	0.0002**	0.0419***	0.0149*	0.0029***	0.0017***
	(0.0000)	(0.0000)	(0.0079)	(0.0077)	(0.0005)	(0.0005)
Size		−0.0008***		0.502***		0.0217***
		(0.0001)		(0.0126)		(0.0008)
Lev		−0.0031***		−0.262***		−0.0123***
		(0.0005)		(0.0547)		(0.0033)
ROA		−0.0011		0.120		0.0168**
		(0.0011)		(0.118)		(0.0072)
ATO		0.0040***		−0.0651**		−0.0072***
		(0.0002)		(0.0257)		(0.0016)
Cashflow		0.0052***		0.0552		0.0000
		(0.0009)		(0.0884)		(0.0054)
REC		0.0184***		1.200***		0.0659***
		(0.0011)		(0.117)		(0.0071)
INV		0.0024***		0.0085		−0.0084*
		(0.0008)		(0.0825)		(0.0051)
FIXED		0.0044***		0.239***		0.0059
		(0.0006)		(0.0673)		(0.0041)
Col		−0.0002		0.0858***		0.0000
		(0.0002)		(0.0219)		(0.0014)
Pgdp		−0.0000		−0.0023		0.0000
		(0.0001)		(0.0128)		(0.0008)
Pop		0.0009		−0.0475		0.0036
		(0.0006)		(0.0654)		(0.0040)
Constant	0.0085***	0.0082***	0.952***	−9.671***	0.0285***	−0.491***
	(0.0013)	(0.0016)	(0.143)	(1.205)	(0.0087)	(0.0741)
Year fixed	Yes	Yes	Yes	Yes	Yes	Yes
Industry fixed	Yes	Yes	Yes	Yes	Yes	Yes
R-squared	0.142	0.167	0.241	0.287	0.245	0.270
*N*	326	326	326	326	326	326

Standard errors in parentheses. ***p < 0.01, **p < 0.05, *p < 0.1.

### 4.3. Moderating analysis

**Figure 2:**
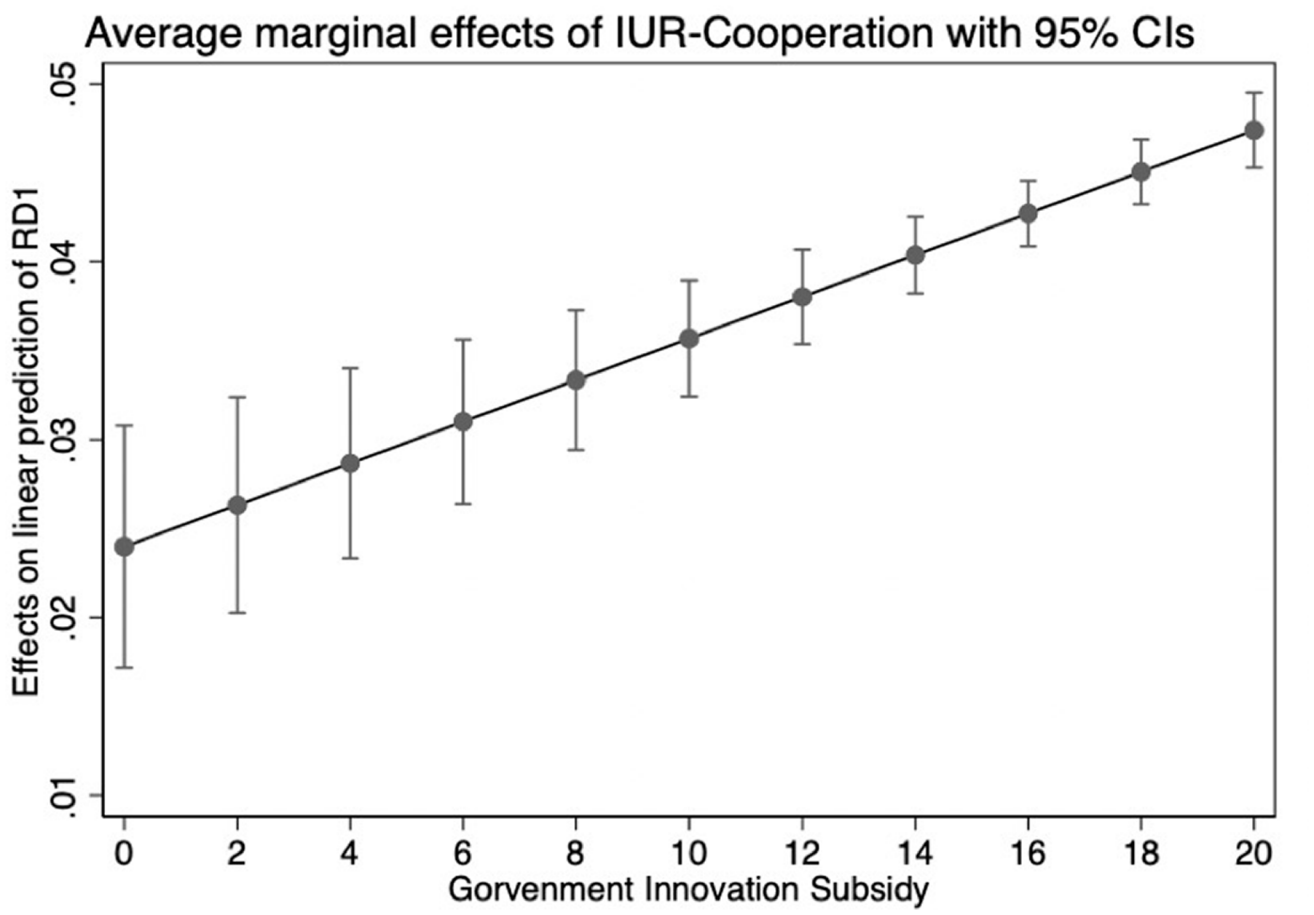
The moderating effect of GIS on RD.

**Figure 3:**
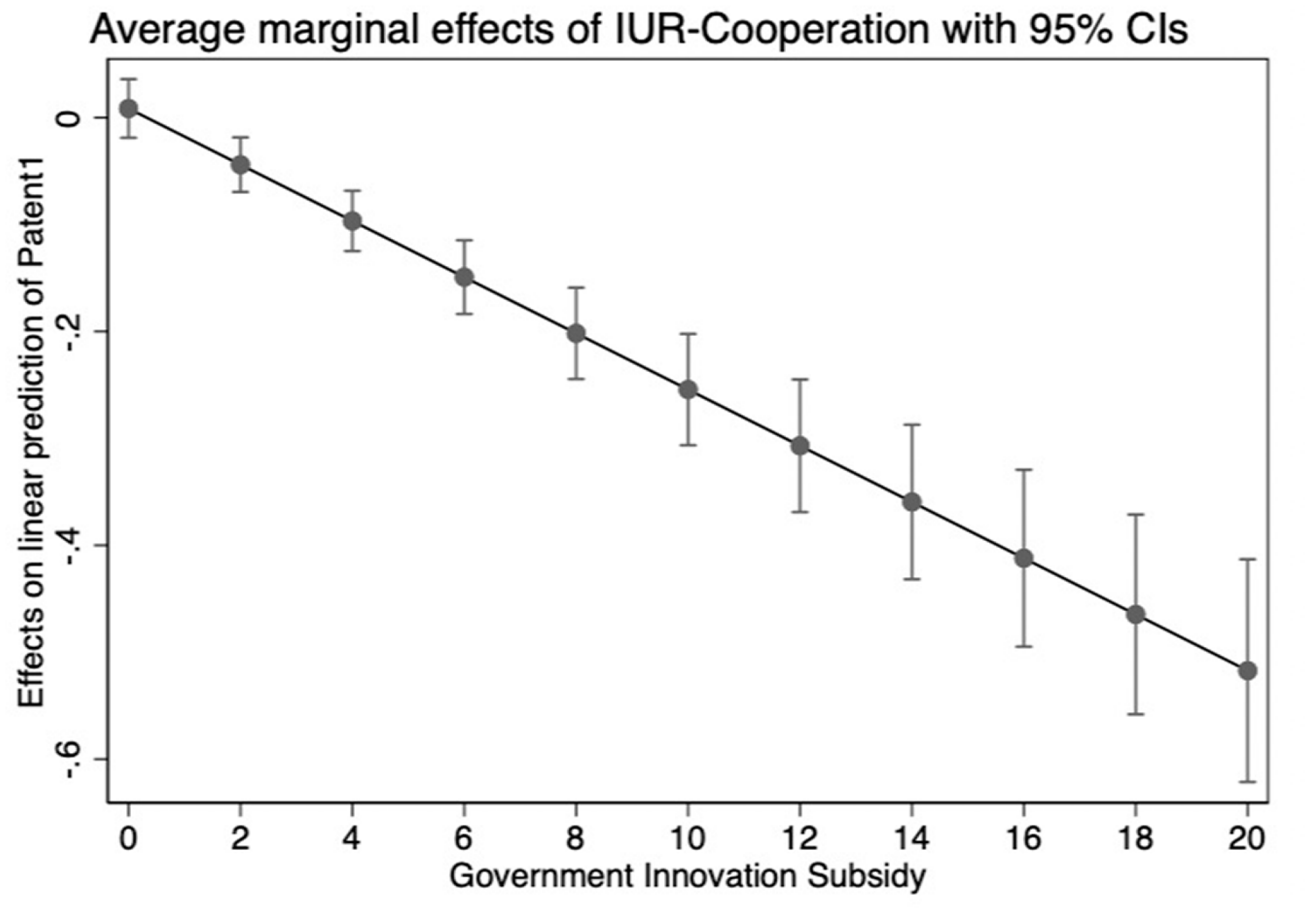
The moderating effect of GIS on patent.

**Figure 4:**
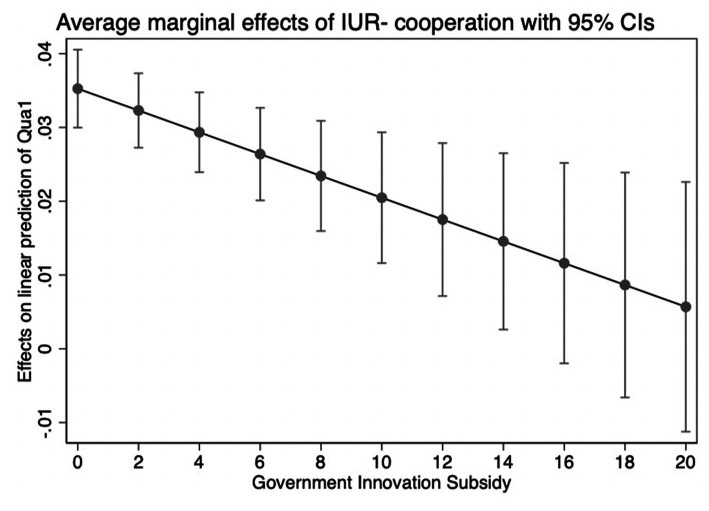
The moderating effect of GIS on quality.

Our evidence so far implies that IUR-cooperation projects can effectively improve innovation performance. Table 4 displays the empirical estimation findings for testing the moderating influence of government innovation subsidies. In column (1), the coefficient of the key interaction term IUR*GIS is positive and significant. This result suggests that the effect of IUR-cooperation projects on innovation inputs is more pronounced in enterprises with higher government innovation subsidies. The results can also be shown in Figure 2. This finding supports H2. In columns (2) and (3), IUR is a variable with significantly positive coefficients. The components associated with GIS are significant, and the interaction term (IUR*GIS) is strongly negative. Thus, government innovation subsidies are not only inefficient at enhancing the outputs of company innovation, but they also have a negative moderating impact, diminishing the importance of IUR-cooperation projects in encouraging the outputs and quality of enterprise innovation. The results can also be shown in Figures 3, 4. Thus, H3 and H4 are also supported.

**Table 4:** Empirical results of government innovation subsidies as moderator.

	(1)	(2)	(3)
Variables	RD1	Patent1	Qua1
IUR	0.0002**	0.0147**	0.0016***
	(0.0000)	(0.0077)	(0.0004)
GIS	0.0001***	0.0202***	0.0012***
	(0.0000)	(0.0016)	(0.0000)
IUR*GIS	−0.0000***	−0.0244***	−0.0012***
	(0.0000)	(0.0018)	(0.0001)
Size	−0.0008***	0.498***	0.0214***
	(0.0001)	(0.0126)	(0.0008)
Lev	−0.0031***	−0.254***	−0.0117***
	(0.0005)	(0.0545)	(0.0034)
ROA	−0.0011	0.130	0.0176**
	(0.0011)	(0.117)	(0.0072)
ATO	0.0040***	−0.0659***	−0.0073***
	(0.0002)	(0.0256)	(0.0016)
Cashflow	0.0052***	0.0659	0.0008
	(0.0009)	(0.0880)	(0.0054)
REC	0.0183***	1.192***	0.0651***
	(0.0011)	(0.116)	(0.0072)
INV	0.0024***	0.0183	−0.0079
	(0.0008)	(0.0822)	(0.0051)
FIXED	0.0043***	0.231***	0.0054
	(0.0006)	(0.0670)	(0.0041)
Col	−0.0002	0.0852***	0.0000
	(0.0002)	(0.0218)	(0.0013)
Pgdp	−0.0000	−0.0011	0.0001
	(0.0001)	(0.0128)	(0.0007)
Pop	0.0009	−0.0346	0.0043
	(0.0006)	(0.0651)	(0.0040)
Constant	0.0081	−9.841***	−0.500***
	(0.0116)	(1.200)	(0.0739)
Year fixed	Yes	Yes	Yes
Industry fixed	Yes	Yes	Yes
R-squared	0.168	0.292	0.275
*N*	326	326	326

Standard errors in parentheses. ***p < 0.01, **p < 0.05, *p < 0.1.

The empirical result that government innovation subsidies in this model do not increase enterprises’ innovation output and efficiency is supported by a substantial empirical research as (75, 76). While China’s industrial and innovation support policies can encourage enterprises to expand the number of innovation inputs, they are not successful in enhancing company innovation output and quality. None of the previous studies address the fact that government innovation subsidies have a negative moderating effect on the influence of IUR-cooperation projects on company innovation output. This study has significant policy implications, including the inadequacy of present innovation support programs in encouraging the outputs and quality of enterprises’ innovation.

### 4.4. Robustness tests

#### 4.4.1. Replacing the dependent variables

This study will conduct robustness tests in the following areas to further assess the dependability of the results of the initial regression (77). RD2, Paten2, and Qua2 are substitutions for RD1, Paten1, and Qua1, respectively. As seen in Table 5, the outcomes are essentially consistent with the main regression.

**Table 5:** Empirical results of replacing the dependent variables.

	(1)	(2)	(3)	(4)	(5)	(6)
Variables	RD2	Patent2	Qua2	RD2	Patent2	Qua2
IUR	0.0005***	0.0193**	0.00206***	0.0005***	0.0191**	0.0020***
	(0.0001)	(0.0091)	(0.0006)	(0.0002)	(0.0091)	(0.0006)
GIS				0.0000***	0.0233***	0.0014***
				(0.0000)	(0.0018)	(0.0001)
IUR*GIS				0.0000***	−0.0274***	−0.0014***
				0.0000	(0.0022)	(0.0001)
Size	0.0009***	0.577***	0.0244***	0.0008***	0.573***	0.0241***
	(0.0002)	(0.0149)	(0.0009)	(0.0003)	(0.0149)	(0.0009)
Lev	−0.0155***	−0.376***	−0.0180***	−0.0154***	−0.367***	−0.0173***
	(0.0011)	(0.0646)	(0.0040)	(0.0011)	(0.0644)	(0.0040)
ROA	−0.0455***	0.0993	0.0186**	−0.0454***	0.111	0.0195**
	(0.0025)	(0.139)	(0.0087)	(0.0025)	(0.139)	(0.0086)
ATO	−0.0106***	−0.0692**	−0.0084***	−0.0106***	−0.0702**	−0.0085***
	(0.0005)	(0.0303)	(0.0019)	(0.0005)	(0.0302)	(0.0019)
Cashflow	−0.0044**	0.0873	0.0011	−0.0043**	0.0997	0.0019
	(0.0018)	(0.104)	(0.0065)	(0.0018)	(0.104)	(0.0065)
REC	−0.0078***	1.402***	0.0768***	−0.0079***	1.392***	0.0758***
	(0.0024)	(0.138)	(0.0086)	(0.0024)	(0.137)	(0.0086)
INV	−0.0093***	−0.0220	−0.0125**	−0.0093***	−0.0111	−0.0119**
	(0.0017)	(0.0975)	(0.0061)	(0.0017)	(0.0971)	(0.0061)
FIXED	0.0019	0.219***	0.0037	0.0018	0.211***	0.0030
	(0.0014)	(0.0795)	(0.0050)	(0.0014)	(0.0792)	(0.0050)
Col	0.0011**	0.102***	−0.0000	0.0010**	0.101***	−0.0000
	(0.0005)	(0.0258)	(0.0016)	(0.0004)	(0.0257)	(0.0016)
Pgdp	−0.0001	−0.0073	−0.0001	−0.0001	−0.0060	−0.0000
	(0.0002)	(0.0152)	(0.0009)	(0.0003)	(0.0151)	(0.0009)
Pop	−0.00078	−0.0884	0.0028	−0.0007	−0.0737	0.0037
	(0.0014)	(0.0772)	(0.0048)	(0.0013)	(0.0769)	(0.0048)
Constant	0.0251	−10.33***	−0.522***	0.0247	−10.53***	−0.533***
	(0.0251)	(1.423)	(0.0888)	(0.0251)	(1.418)	(0.0886)
Year fixed	Yes	Yes	Yes	Yes	Yes	Yes
Industry fixed	Yes	Yes	Yes	Yes	Yes	Yes
R-squared	0.166	0.278	0.263	0.166	0.283	0.268
*N*	326	326	326	326	326	326

Standard errors in parentheses. ***p < 0.01, **p < 0.05, *p < 0.1.

#### 4.4.2. Instrumental variable method

We carried out an instrumental variable analysis to allay the endogeneity worry brought on by omitted variables. As the instrumental variables (IV), designated by IUR_PRO and IUR _PRO_IND, respectively, we selected the average IUR-cooperation projects of the other enterprises situated in the same province and the average IUR-cooperation projects of the other firms located in the same province and belonging to the same industry. The method used as Zhou et al. (78) and Kanama and Nishikawa (79). The rationale behind establishing the number of tool variables in this manner is that the level of IUR-cooperation projects is obviously influenced by the features of regions and industries due to the various innovation resources and innovation requirements. Consequently, the total of industry-academic-research-cooperation variables at the regional and industry levels is anticipated to have a significant impact on the scope of enterprises’ participation in IUR-cooperation. Regional and firm-level industry-university cooperation is unlikely to have a systematic effect on the innovation quality of individual enterprises. IUR _PRO and IUR _PRO_IND can therefore be used as the best instrumental variables for an industry-university partnership because they are consistent with the chosen standard of a valid instrumental variable.

Based on Jin and Wu’s (80) method from 2021, we adopted a two-stage approach for this analysis. The results can be found in Table 6. In the first stage, we performed a regression of IUR-cooperation projects on IV. In the second stage, we replaced the IUR-cooperation projects index in the full model with the arithmetic mean of the fitted value from the first stage. Table 6 displays the estimated outcomes. In columns (1) and (5), the results of the first stage indicate that both instrumental variables have a significant positive correlation with IUR cooperation. The coefficients of IUR-cooperation projects in columns (2)–(4) and (6)–(8) are all positive and statistically significant. The outcomes are consistent with our initial findings.

**Table 6:** Empirical results of instrumental variable method.

	IV = IUR _PRO				IV = IUR _PRO_IND			
	(1)	(2)	(3)	(4)	(5)	(6)	(7)	(8)
Variables	IUR	RD1	Patent1	Qua1	IUR	RD1	Patent1	Qua1
IV	0.0981***				0.0985***			
	(0.0003)				(0.0015)			
IUR		0.0058***	0.368***	0.0172***		0.00213***	0.150***	0.0064***
		(0.0046)	(0.0401)	(0.0023)		(0.0002)	(0.0226)	(0.0013)
Size	0.0726***	−0.0117***	0.565***	0.0227***	0.0694***	−0.0009***	0.581***	0.0236***
	(0.0057)	(0.0058)	(0.0081)	(0.0005)	(0.0055)	(0.0000)	(0.0076)	(0.0004)
Lev	−0.3781***	−0.0643***	−0.348***	−0.0158***	−0.38231***	−0.0079***	−0.436***	−0.0201***
	(0.0065)	(0.0006)	(0.0529)	(0.0030)	(0.0012)	(0.0005)	(0.0502)	(0.0029)
ROA	−0.0006	0.0161***	0.596***	0.0396***	−0.0978	0.0159***	0.581***	0.0389***
	(0.1061)	(0.0019)	(0.141)	(0.0079)	(0.0016)	(0.0018)	(0.138)	(0.0078)
ATO	−0.1025***	0.0042***	0.138***	0.00334***	−0.0979***	0.0044***	0.118***	0.00235**
	(0.0140)	(0.0002)	(0.0205)	(0.0012)	(0.0131)	(0.0002)	(0.0198)	(0.0011)
Cashflow	−0.1652**	0.0186***	0.719***	0.0309***	−0.1422	0.0193***	0.756***	0.0327***
	(0.0869)	(0.0001)	(0.118)	(0.0066)	(0.0840)	(0.0013)	(0.115)	(0.0065)
REC	1.292***	0.0172***	1.742***	0.0996***	1.2272***	0.0222***	2.022***	0.113***
	(0.0661)	(0.0012)	(0.105)	(0.0059)	(0.0646)	(0.0011)	(0.0948)	(0.0053)
INV	−0.0020	0.026***	0.0152	−0.0013	−0.0231	0.0025***	0.0103	−0.00161
	(0.0554)	(0.0007)	(0.0730)	(0.0042)	(0.0534)	(0.0006)	(0.0713)	(0.0041)
FIXED	−0.2581***	−0.0043***	−0.672***	−0.0395***	−0.2121***	−0.0053***	−0.737***	−0.0427***
	(0.0437)	(0.0006)	(0.0603)	(0.0034)	(0.0352)	(0.00057)	(0.0583)	(0.0034)
Col	0.0104**	0.0102***	0.0719***	0.00280***	0.0094**	0.0016***	0.0834***	0.0834***
	(0.0053)	(0.0002)	(0.0136)	(0.0008)	(0.0045)	(0.0001)	(0.0024)	(0.0024)
Pgdp	−0.0282***	0.0027***	0.144***	0.00753***	−0.0192***	0.0010***	0.0648***	0.00245***
	(0.0046)	(0.0001)	(0.0126)	(0.0007)	(0.0099)	(0.0001)	(0.0132)	(0.0008)
Pop	−0.0161*	0.0004	0.3681***	0.0172***	0.0231*	0.0037	0.175***	0.0091***
	(0.0009)	(0.000)	(0.0401)	(0.0022)	(0.0009)	(0.0048)	(0.0115)	(0.0006)
Constant	0.2543**	−0.0193***	−0.522***	0.565***	0.2543**	−0.533***	−15.97***	−0.693***
	(0.0025)	(0.003)	(0.0888)	(0.0081)	(0.0025)	(0.0886)	(0.3115)	(0.0181)
Year fixed	Yes	Yes	Yes	Yes	Yes	Yes	Yes	Yes
Industry fixed	Yes	Yes	Yes	Yes	Yes	Yes	Yes	Yes
R-squared	0.328	0.457	0.445	0.423	0.283	0.268	0.472	0.440
*N*	326	326	326	326	326	326	326	326

Standard errors in parentheses. ***p < 0.01, **p < 0.05, *p < 0.1.

## 5. Discussion

### 5.1. Discussion and conclusion

This study seeks to investigate whether and how Chinese biopharmaceutical firms leverage IUR-cooperation projects to enhance their innovation performance. To achieve this goal, we manually gathered data on IUR-cooperation projects from disclosures made by 326 listed Chinese biopharmaceutical firms between 2007 and 2021. We then applied a fixed effect model to assess the impact. Specifically, our findings reveal that IUR-cooperation projects have significantly improved the innovation performance of listed Chinese biopharmaceutical companies. They have led to a 0.002 percentage point increase in innovation inputs and a 0.0147 percentage point boost in innovation quality. These results indicate that IUR-cooperation projects not only augment the innovative input of biopharmaceutical enterprises but also contribute to the quality and quantity of their innovation. These findings extend empirical research on the influence of IUR-cooperation projects on the innovation performance of biopharmaceutical firms from the initial stages of collaboration, an aspect often overlooked in existing literature. Furthermore, our research demonstrates that government innovation subsidies positively impact the relationship between IUR-cooperation projects and innovative input, a finding consistent with Bozeman and Gaughan’s (81). However, government innovation subsidies have a contrasting effect on the link between IUR-cooperation projects and innovation outputs and quality, leading to a negative moderation. This contrasts with the findings of Zhang, Yuan and Wang (82), who examined the diversity of partners involved in IUR-cooperation.

### 5.2. Theoretical implications of the study

In this article, the independent variables are quantified by assessing the corporative projects between listed companies and academia, unlike literature focus on paper and patents (21). These projects are the beginning of the inception of knowledge integrate with enterprises, universities, and research institutions. Thus, quantifying the cooperative aspect of the knowledge generation stage within the knowledge-based theory. The dependent variable under investigation pertains to the research and development performance of these listed companies, quantifying their knowledge utilization status. This provides evidence for the impact of IUR cooperation on knowledge theory. Furthermore, we introduce a consideration for the quality of innovation. The primary objective of IUR cooperation is to facilitate the integrate the cutting-edge technology and scientific advancements from universities and research institutes to enterprises, thus aiding them in achieving high-quality innovation. In line with the goals of IUR cooperation, we employ exploitation innovation (61) as a metric for evaluating innovation quality, a novel approach in the field. Measuring innovation quality using the breadth-of-knowledge method is a seldom explored aspect in previous research. Notably, our study recognizes the government’s pivotal role as a stakeholder in IUR cooperation, involving multiple parties. However, prior research often approaches the impact of policy trends from the perspective of universities. Zhang et al. (83) based on a novel sample of the China Academy of Sciences (CAS), collect data from 1978 to 2015 and test the government policy may have an influence on cooperation structures. In contrast, our analysis focuses on government subsidies for innovation as a conditional variable, providing a more targeted examination that accurately acknowledges the government’s role in the context of cooperative innovation. This approach aligns with our objective to gain a deeper understanding of the government’s influence in this arena.

### 5.3. Practical implication

For administrators, our study underscores the importance of fostering intensified interactions and cooperation between industries, educational institutions, and other entities. Such cooperation should be structured to ensure that both parties can grow in alignment with the necessary supporting infrastructure and more. The synergy between businesses, academia, and research institutions plays a pivotal role in fostering innovation and development. Our findings also lead to the conclusion that government innovation policies cannot solely rely on government subsidies, as this approach may distort their effectiveness. In the realm of innovation policy, evaluation criteria such as “input but not output” and “quantity but not quality” should be reconsidered. Government innovation subsidies should aim to support the synergy between firms’ innovation performance and IUR-cooperation while alleviating the costs associated with identifying collaborators, facilitating communication, and assessing potential risks. The government’s role should encompass the establishment of a platform for IUR-cooperation initiatives, along with the optimization of the innovation market environment and legal framework.

### 5.4. Future research

The following outlines the potential avenues for future research: (1) Businesses aspire to cooperate and innovate, aiming to secure new products and technologies and subsequently penetrate new markets. Our current preference for assessing innovation indicators involves the examination of patent data. However, in our future research, we may explore the adoption of more market-oriented indicators. (2) The majority of our research has concentrated on publicly traded pharmaceutical companies. These listed firms tend to be larger in size, yet their inclination and capacity for collaboration may not be as strong as that of smaller businesses. However, information about non-listed companies is often not readily accessible or transparent. In the future, should we gain access to suitable data, we could consider analyzing samples from SMEs.

## Data availability statement

The original contributions presented in the study are included in the article/Supplementary material, further inquiries can be directed to the corresponding author.

## Author contributions

YX: Conceptualization, Data curation, Formal analysis, Investigation, Methodology, Software, Writing – original draft. YJ: Funding acquisition, Project administration, Supervision, Visualization, Writing – review & editing.

## Supplementary material

The Supplementary material for this article can be found online at:

https://www.frontiersin.org/articles/10.3389/fpubh.2023.1271364/full#supplementary-material
