# Supplementary figures and images for: The impact of industry-university-research projects on biopharmaceutical companies’ innovation performance: moderating roles of government subsidies for innovation
Source: Front Public Health. 2023 Nov 15;11:1271364. doi: 10.3389/fpubh.2023.1271364 (PMC10684675; doi:10.3389/fpubh.2023.1271364)

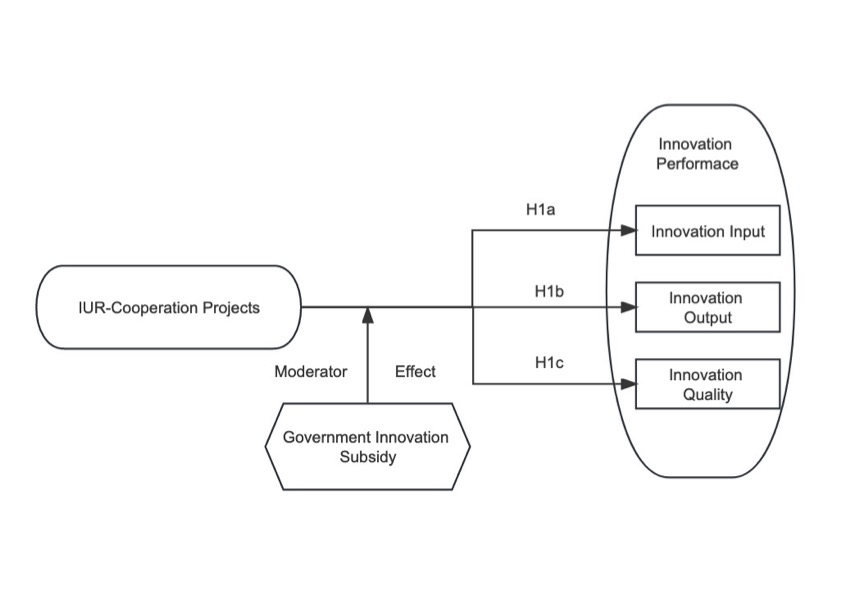

Supplement: Supplementary file 1 [file Data_Sheet_1.ZIP › Supplementary Material Presentation/Figure 1.jpg]

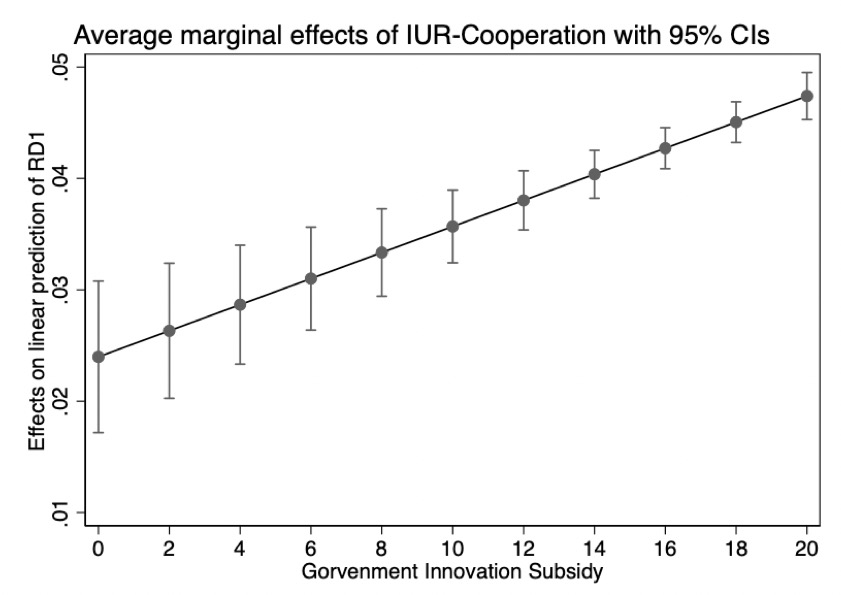

Supplement: Supplementary file 1 [file Data_Sheet_1.ZIP › Supplementary Material Presentation/Figure 2.jpg]

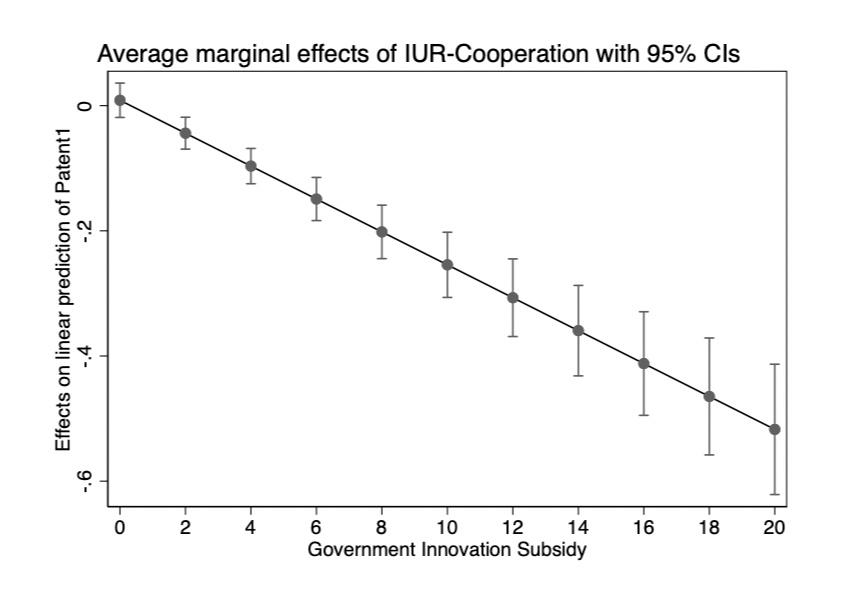

Supplement: Supplementary file 1 [file Data_Sheet_1.ZIP › Supplementary Material Presentation/Figure 3.jpg]

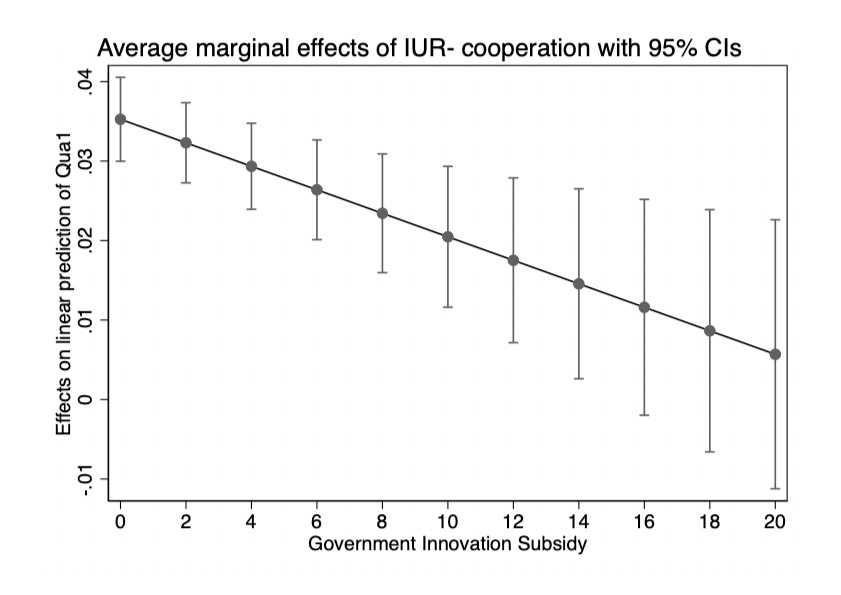

Supplement: Supplementary file 1 [file Data_Sheet_1.ZIP › Supplementary Material Presentation/Figure 4.jpg]
